# Adult-Onset Still’s Disease in a 49-Year-Old Female in the UAE: A Case Report and Literature Review

**DOI:** 10.7759/cureus.87858

**Published:** 2025-07-13

**Authors:** Nagwa M Ali, Mohammed O Ibrahim, Osama M Mohamed

**Affiliations:** 1 Rheumatology, Mediclinic Airport Road Hospital, Abu Dhabi, ARE; 2 Clinical Sciences, College of Medicine, Mohammed Bin Rashid University of Medicine and Health Sciences, Dubai, ARE; 3 Family Medicine, SEHA Clinics, Abu Dhabi Health Services Company (SEHA), Abu Dhabi, ARE

**Keywords:** adult-onset still’s disease, biological therapy, hyperferritinemia, salmon-pink rash, yamaguchi criteria

## Abstract

Adult-onset Still’s disease (AOSD) is a rare autoinflammatory disorder characterized by high spiking fevers, salmon-colored rash, arthritis, and hyperferritinemia. It often mimics infections or autoimmune conditions, leading to diagnostic delays. We report a case of a 49-year-old female who presented with fever, fatigue, arthralgia, evanescent rash, and elevated inflammatory markers. Extensive infectious and autoimmune workups were negative. Her serum ferritin exceeded 16,000 ng/mL. She fulfilled the Yamaguchi criteria for AOSD, and treatment with corticosteroids followed by tocilizumab resulted in marked clinical improvement. This is, to our knowledge, the first documented case of AOSD from the UAE. It underscores the importance of ferritin in diagnosis and disease monitoring and highlights the efficacy of interleukin (IL)-6 inhibition in management.

## Introduction

Adult-onset Still's disease (AOSD) is a rare autoinflammatory disorder characterized by a constellation of symptoms, including high-grade fever, arthralgia or arthritis, and a distinctive evanescent rash that often coincides with fever spikes. The disease predominantly affects young adults, with a slightly higher prevalence in women, and can manifest in monocyclic, polycyclic, or chronic forms, with chronic cases primarily affecting the joints [1]. The pathogenesis of AOSD involves the activation of the innate immune response, characterized by increased secretion of cytokines such as Interleukin (IL)-1, IL-6, and IL-18, and is often accompanied by elevated serum ferritin levels, which reflect systemic inflammation [2]. Diagnosis is challenging due to the overlap of symptoms with other conditions, necessitating the exclusion of infections, neoplasms, and other rheumatological diseases. The Yamaguchi criteria, which are among the most widely used diagnostic tools for AOSD, include major features such as high spiking fever, arthralgia or arthritis, a transient salmon-pink rash, and leukocytosis with neutrophil predominance. Minor criteria include sore throat, lymphadenopathy, hepatosplenomegaly, abnormal liver function tests, and negative tests for rheumatoid factor and antinuclear antibodies. A diagnosis typically requires at least five criteria, including two major ones, after excluding infections, malignancies, and other rheumatologic conditions [2, 3]. Treatment typically involves glucocorticoids and disease-modifying antirheumatic drugs (DMARDs) like methotrexate and ciclosporin to reduce steroid dependency. In cases of high disease activity, biologics such as the IL-1 receptor antagonist anakinra, the IL-1β antibody canakinumab, and the IL-6 receptor blocker tocilizumab are employed. However, their use may be limited by local drug licensing policies [4]. AOSD can present with severe complications, including perimyocarditis, macrophage activation syndrome (MAS), and rare neurological manifestations like acute encephalitis [5]. The disease's rarity and nonspecific early symptoms often lead to misdiagnosis and delayed treatment, underscoring the need for heightened clinical awareness and thorough differential diagnosis, especially in cases of fever of unknown origin [6]. Despite the challenges in diagnosis and management, early recognition and appropriate treatment can significantly improve patient outcomes [7].

AOSD's epidemiology shows significant regional variation. In Western Australia, the incidence of AOSD is reported at 0.22 per 100,000, with a point prevalence of 2.4 per 100,000 as of 2013 [8]. In contrast, studies from Japan and France report annual incidences of 0.22 and 0.16 per 100,000, respectively, while Northern Norway reports a higher incidence of 0.4 per 100,000 [9, 10]. The prevalence in Northern Norway increased from 3.4 to 6.9 per 100,000 over a decade [11]. Although these figures provide a reference point, the specific epidemiological data for AOSD in the UAE remains unreported in the current literature. The UAE has been studied for other conditions, such as transverse myelitis and neuromyelitis optica spectrum disorders, with specific prevalence and incidence rates provided for these conditions [12]. However, without direct studies or data on AOSD in the UAE, it is challenging to draw precise conclusions about its epidemiology in this region. 

To date, no published case reports on AOSD in the UAE have been found in indexed databases, including PubMed, MEDLINE, Scopus, and Web of Science. To our knowledge, this is the first case of AOSD reported in Abu Dhabi, emphasizing the importance of clinician awareness and timely identification of this uncommon disorder in the region. The lack of regional data underlines the need for scholarly contributions from underrepresented geographic areas. This case is significant as it shows a classic yet diagnostically complex presentation of AOSD, characterized by systemic symptoms, markedly elevated ferritin levels, and a significant response to IL-6 inhibition [13]. It demonstrates the utility of repeated inflammatory markers, comprehensive autoimmune panels, and imaging in ruling out differential diagnoses [13]. Additionally, the case outlines the treatment approach in the UAE setting, including the use of tocilizumab, which is supported by current international guidelines [14]. 

The objective of this report is to raise clinical awareness of AOSD among healthcare professionals in the UAE, to demonstrate the diagnostic utility of ferritin and cytokine profiling in systemic febrile syndromes, and to contribute to the limited body of literature from this region. It also provides a framework for managing refractory cases using targeted biological therapy. 

## Case presentation

**Figure 1:**
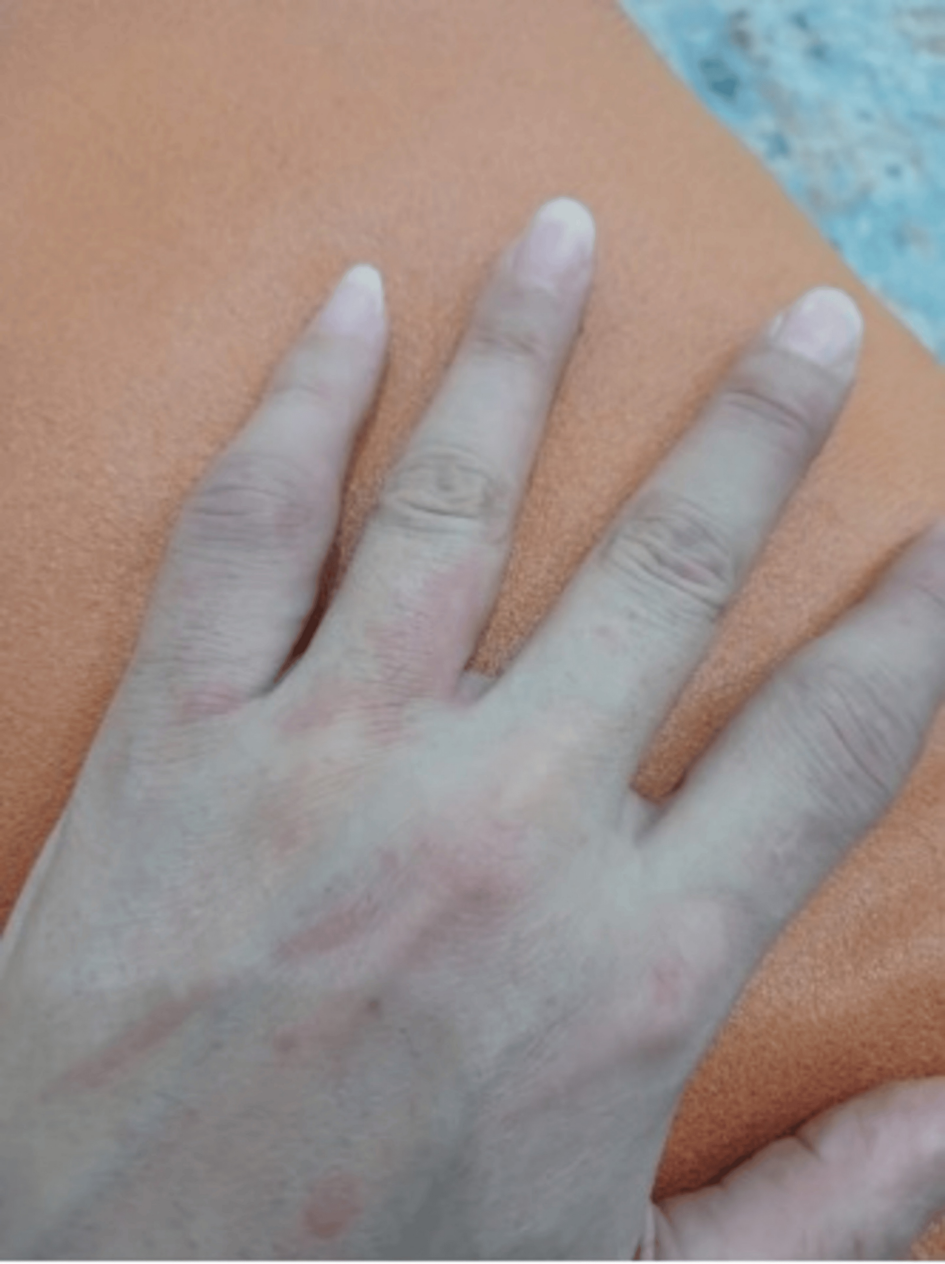
Salmon-pink maculopapular rash over the dorsum of the hand, typical of adult-onset Still’s disease and noted to intensify with febrile episodes

**Figure 2:**
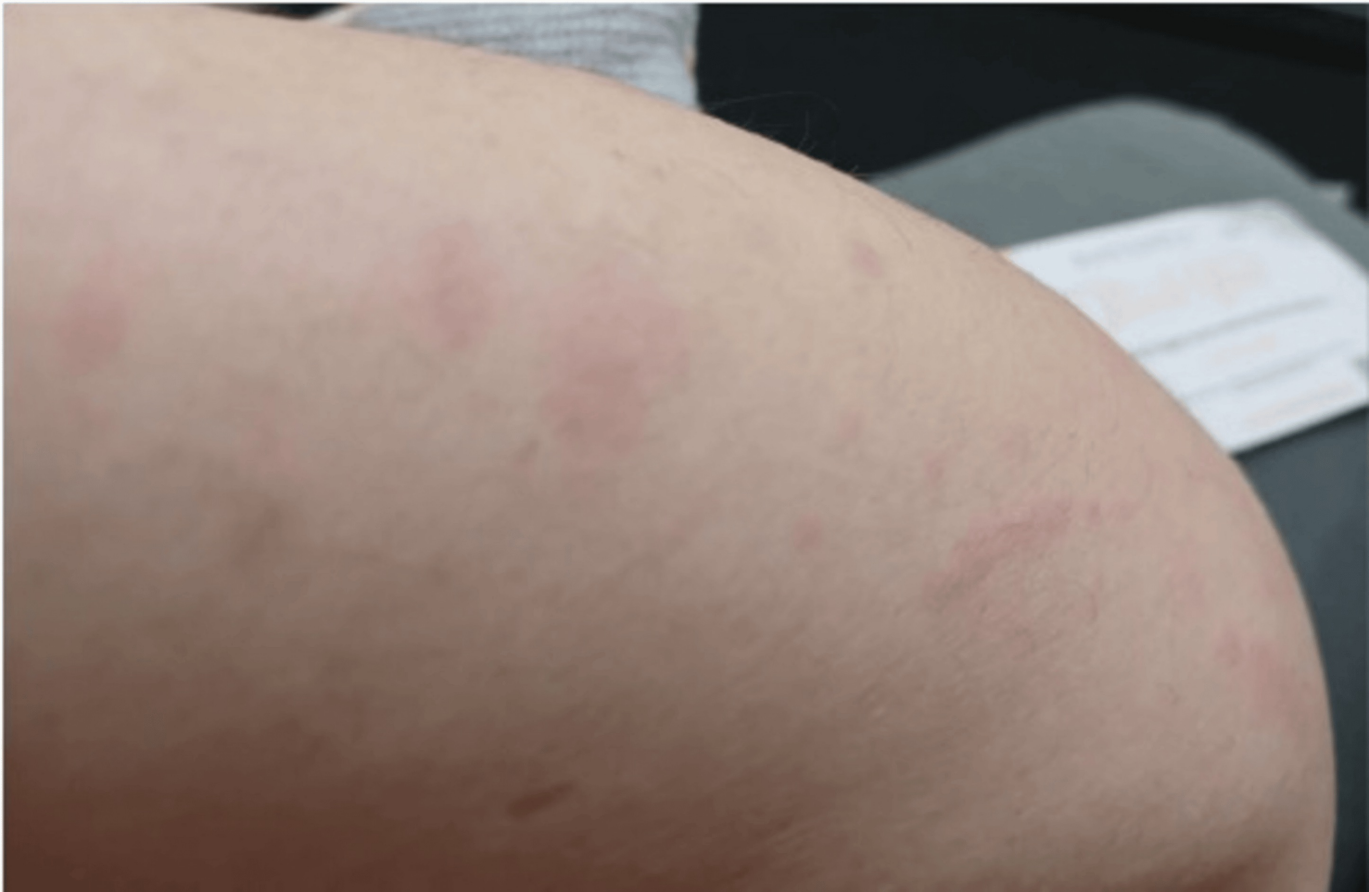
Intermittent salmon-colored maculopapular rash on the forearm, observed to flare during fever spikes in adult-onset Still’s disease

A 49-year-old female with no known past medical history presented to the rheumatology clinic in September 2024 with a constellation of symptoms, including generalized fatigue, weight loss, dry eyes, joint stiffness, and polyarthralgia involving the knees, fingers, and hips. She also reported a salmon-pink maculopapular rash that appeared intermittently and was exacerbated by fever, as shown in Figures 1, 2.

Her family history was notable for systemic lupus erythematosus (SLE), fibromyalgia, and rheumatoid arthritis. On physical examination, the patient appeared pale and lethargic, with evidence of dry skin. The musculoskeletal assessment indicated tenderness at the medial joint line in both knees, tenderness in the ankles, a reduced straight leg raise bilaterally, limited active range of motion in the joints, and an abnormal gait pattern. Initial laboratory investigations revealed markedly elevated inflammatory markers, with a serum ferritin level of 16,286 µg/L and an erythrocyte sedimentation rate (ESR) of 80 mm/hr. The hematologic analysis revealed anemia (hemoglobin 8.6 g/dL), leukocytosis (white blood cell count 22.7 × 10⁹/L), and thrombocytosis (platelet count 538 × 10⁹/L). Autoimmune serologies were unremarkable, with negative antinuclear antibody (ANA), antineutrophil cytoplasmic antibodies (ANCA), and anti-cyclic citrullinated peptide (CCP) antibodies. A comprehensive summary of relevant serum investigations is presented in Table 1. 

**Table 1:** Serum laboratory investigations at presentation LDH: Lactate Dehydrogenase; CCP: Cyclic Citrullinated Peptide; DNA: Deoxyribonucleic Acid; SCL: Scleroderma; SSA: Sjögren’s Syndrome Antigen A;  SSB: Sjögren’s Syndrome Antigen B; RNP: Ribonucleoprotein;   EGFR: Estimated Glomerular Filtration Rate; ANA: Antinuclear Antibody; ANCA: Antineutrophil Cytoplasmic Antibodies; ESR: Erythrocyte Sedimentation Rate.

Test	Result	Reference Range
LDH	331 U/L	< 250 U/L
C3 Complement	159 mg/dL	90–180 mg/dL
C4 Complement	36.6 mg/dL	10–40 mg/dL
Anti-TG (Thyroglobulin Ab)	18.3 IU/mL	< 115 IU/mL
Anti-CCP	< 8 U/mL	< 17 U/mL
Lupus Anticoagulant Panel	Negative	Negative
Cardiolipin IgM	2.4 MPL-U/mL	< 7 MPL-U/mL
Cardiolipin IgG	2.3 GPL-U/mL	< 10 GPL-U/mL
Antiphospholipid IgG	3.2 GPL-U/mL	< 10 GPL-U/mL
Antiphospholipid IgM	2.8 MPL-U/mL	< 10 MPL-U/mL
Beta-2-glycoprotein-1 IgG	2.1 U/mL	< 5 U/mL
Beta-2-glycoprotein-1 IgM	2.4 U/mL	< 5 U/mL
Anti-dsDNA	20.1 U/mL	< 25 U/mL
Anti-JO1	Negative	Negative
Anti-SCL-70 Abs	Negative	Negative
Anti-Smith Abs	Negative	Negative
Anti-SSA	Negative	Negative
Anti-SSB	Negative	Negative
Anti-RNP	Negative	Negative
EGFR	93 mL/min/1.73 m²	< 60 mL/min/1.73 m²
Folate	17 ng/mL	4.4–31 ng/mL
ANA	Negative	Negative
ANCA	Negative	Negative
Ferritin	16,286 µg/L	20–300 µg/L
ESR	80 mm/hr	0–20 mm/hr
Hemoglobin	8.6 g/dL	12–16 g/dL
White Blood Cells	22.7 ×10⁹/L	4–11 ×10⁹/L
Platelet Count	538 ×10⁹/L	150–400 ×10⁹/L

**Figure 3:**
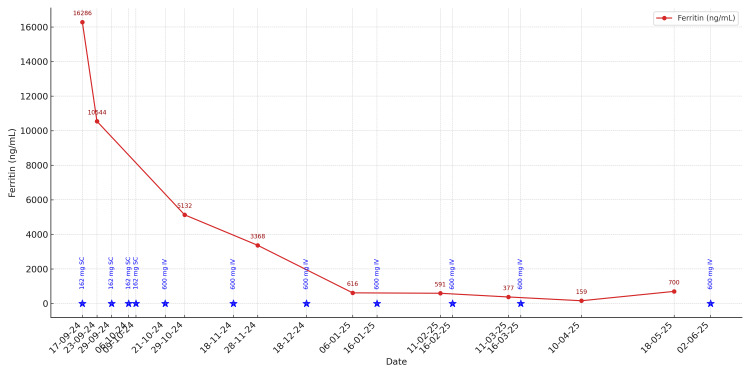
Trend of serum ferritin levels over time with Tocilizumab dose markers (blue stars represent dosing events annotated with dose and route)

The patient met the Yamaguchi criteria for AOSD, and other diagnoses, including infections, malignancy, and connective tissue diseases, were excluded. She was initially treated with intramuscular methylprednisolone and started on subcutaneous Tocilizumab 162 mg weekly, later escalated to 600 mg IV monthly. Despite initial steroid responsiveness, disease flares occurred upon tapering, prompting the addition of azathioprine and later methotrexate. Over the subsequent months, her inflammatory markers steadily improved, with ferritin levels declining to 159 µg/L and ESR to 25 mm/hr by April 2025. The trend of serum ferritin levels over time, in relation to Tocilizumab administration, is illustrated in Figure 3. Each Tocilizumab dose is marked by a blue star positioned along the timeline, annotated with the corresponding dose and route of administration (subcutaneous or intravenous). The graph demonstrates a clear temporal association between biological therapy and the progressive decline in ferritin levels, highlighting the role of Tocilizumab in controlling systemic inflammation and the utility of serial ferritin monitoring as a marker of disease activity in AOSD.

**Figure 4:**
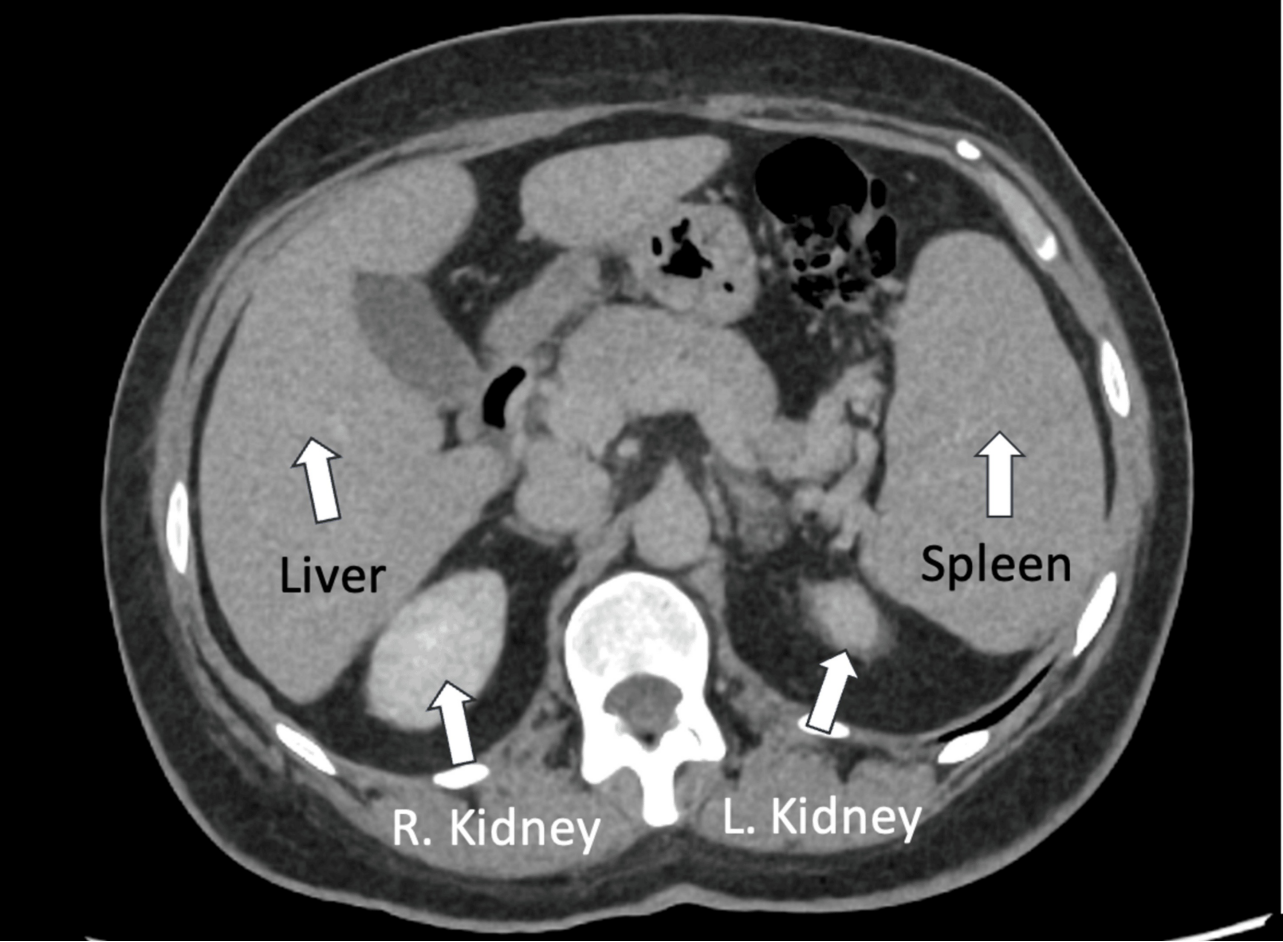
Axial CT abdomen demonstrating spleen mildly enlarged in size (14.7 cm span)

**Figure 5:**
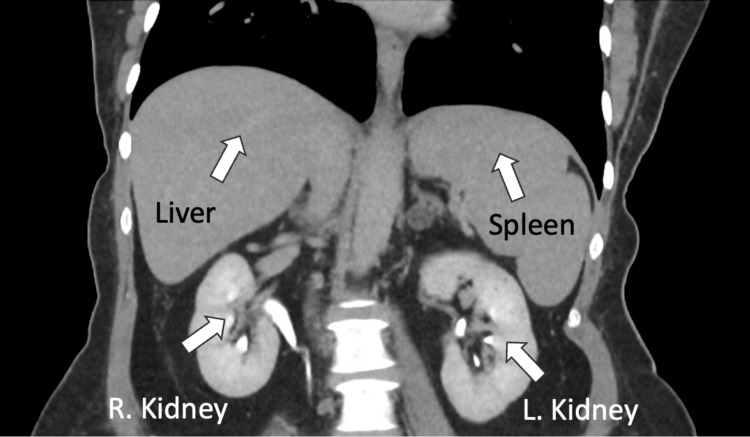
Coronal CT abdomen showing mildly enlarged spleen

Imaging studies, such as abdominal CT, showed splenomegaly and a small hepatic hemangioma but no evidence of malignancy. The CT abdomen demonstrated mild splenomegaly, with the spleen measuring 14.7 cm in span. Representative axial and coronal CT images (Figures 4, 5) clearly show the mildly enlarged spleen and its relation to adjacent structures, including the left kidney.

The patient remains under regular follow-up and continues to receive tocilizumab infusions with improved clinical and laboratory outcomes.

## Discussion

AOSD is a rare, systemic autoinflammatory disorder with significant diagnostic complexity, especially in regions where it is underreported or unfamiliar to practitioners. The patient described in this case exhibited classical features of AOSD, including quotidian fever, evanescent salmon-pink rash, arthralgia, leukocytosis with neutrophilic predominance, and markedly elevated ferritin levels, fulfilling the Yamaguchi criteria for diagnosis [3]. The exclusion of infectious, neoplastic, and other autoimmune etiologies through extensive laboratory and imaging evaluations was instrumental in confirming the diagnosis. Elevated serum ferritin, in particular, has been recognized as a hallmark of AOSD and may correlate with disease activity [15]. This patient’s laboratory findings, including marked hyperferritinemia, elevated ESR, anemia, thrombocytosis, and neutrophilic leukocytosis, mirror those commonly reported in AOSD, further reinforcing the clinical diagnosis [15]. 

The treatment plan adhered to international standards and reflected current shifts in the management of AOSD. Initial corticosteroid therapy led to partial clinical improvement, as expected with first-line treatment recommendations [2]. However, steroid tapering led to disease flares, prompting the addition of disease-modifying antirheumatic drugs (DMARDs) such as methotrexate and azathioprine, which are commonly used to minimize steroid dependence in chronic or refractory disease [2]. Treatment escalation was guided by the recurrence of systemic symptoms and persistently high inflammatory markers, such as ferritin and ESR, particularly during the period of corticosteroid tapering.

Biologic therapy was initiated with tocilizumab, an IL-6 receptor antagonist, due to persistent systemic symptoms and elevated inflammatory markers. Tocilizumab has demonstrated efficacy in patients with refractory AOSD and is endorsed by the 2022 European Alliance of Associations for Rheumatology (EULAR) and American College of Rheumatology (ACR) guidelines for cases unresponsive to conventional therapy [16]. In this case, the patient experienced both clinical improvement and a gradual reduction in ferritin and ESR following tocilizumab treatment. The decision to switch from subcutaneous to intravenous delivery was made after careful consideration of disease progression and therapeutic response. This case also highlights the logistical and clinical considerations involved in administering biologics in the UAE, where accessibility and insurance approval can influence therapeutic decisions. 

The presence of a family history of autoimmune diseases added complexity to the diagnostic process. While ANA positivity is rare in AOSD, the patient's seronegativity supported the AOSD diagnosis rather than a flare of lupus or overlap syndrome [17]. Additionally, splenomegaly observed on imaging, though nonspecific, is a common feature in systemic inflammatory diseases and supports the clinical picture of AOSD [1].

To the best of our knowledge, this is the first detailed and indexed case report of AOSD in the UAE, highlighting the need for increased awareness of the condition in this region. While another non-indexed report described AOSD in the context of post-COVID inflammation, our case represents the classic presentation of AOSD, characterized by typical clinical features, imaging findings, and response to biologic therapy [18]. Given the scarcity of regional data and reports, sharing cases like this plays a key role in guiding local clinicians on how to identify and manage rare systemic inflammatory conditions. 

## Conclusions

This case highlights the diagnostic and therapeutic challenges associated with adult-onset Still’s disease, particularly in regions where the condition is underrecognized. Our patient’s clinical course highlights the importance of considering AOSD in the differential diagnosis of prolonged fever, typical rash and systemic inflammation, as well as the value of ferritin as a marker of disease activity. The favorable outcome achieved with tocilizumab in this case supports the growing evidence for biologic agents as an effective option in the management of treatment-resistant AOSD. Sharing what appears to be the first indexed report of AOSD from the UAE, we hope to promote greater recognition of this uncommon condition among healthcare professionals in the region and contribute to the broader medical literature on autoinflammatory diseases. Further studies are warranted to better define the epidemiology, diagnostic strategies, and optimal treatment pathways for AOSD in this region. 
